# Nanostructures Derived from Starch and Chitosan for Fluorescence Bio-Imaging

**DOI:** 10.3390/nano6070130

**Published:** 2016-07-05

**Authors:** Yinxue Zu, Jingran Bi, Huiping Yan, Haitao Wang, Yukun Song, Bei-Wei Zhu, Mingqian Tan

**Affiliations:** 1 Liaoning Key Laboratory of Food Biological Technology, School of Food Science and Technology, Dalian Polytechnic University, Qinggongyuan 1, Ganjingzi District, Dalian 116034, China; zuyinxue123@163.com (Y.Z.); bijingran125@163.com (J.B.); yanhp@seashine.cn (H.Y.); wanght6@126.com (H.W.); songyukun@126.com (Y.S.); zhubeiwei@163.com (B.-W.Z.); 2 School of Food and Biological Engineering, Jiangsu University, Zhenjiang 212013, China

**Keywords:** polysaccharide nanostructures, fluorescent materials, quantum yield, cell imaging, fish imaging

## Abstract

Fluorescent nanostructures (NSs) derived from polysaccharides have drawn great attention as novel fluorescent probes for potential bio-imaging applications. Herein, we reported a facile alkali-assisted hydrothermal method to fabricate polysaccharide NSs using starch and chitosan as raw materials. Transmission electron microscopy (TEM) demonstrated that the average particle sizes are 14 nm and 75 nm for starch and chitosan NSs, respectively. Fourier transform infrared (FT-IR) spectroscopy analysis showed that there are a large number of hydroxyl or amino groups on the surface of these polysaccharide-based NSs. Strong fluorescence with an excitation-dependent emission behaviour was observed under ultraviolet excitation. Interestingly, the photostability of the NSs was found to be superior to fluorescein and rhodamine B. The quantum yield of starch NSs could reach 11.12% under the excitation of 360 nm. The oxidative metal ions including Cu(II), Hg(II)and Fe(III) exhibited a quench effect on the fluorescence intensity of the prepared NSs. Both of the two kinds of the multicoloured NSs showed a maximum fluorescence intensity at pH 7, while the fluorescence intensity decreased dramatically when they were put in an either acidic or basic environment (at pH 3 or 11). The cytotoxicity study of starch NSs showed that low cell cytotoxicity and 80% viability was found after 24 h incubation, when their concentration was less than 10 mg/mL. The study also showed the possibility of using the multicoloured starch NSs for mouse melanoma cells and guppy fish imaging.

## 1. Introduction

Novel nanostructures (NSs) with multicoloured fluorescence have attracted much attention because of their potential biological applications including molecular labelling, contrast medium, sensors and image-guided drug release [1,2,3]. Traditional semiconductor quantum dots (QDs) have been widely used for bio-analysis in the last decade because of their good fluorescence characteristics; however, they are constituted of toxic heavy metals and suffer from serious safety concerns and environmental risks [1]. Novel fluorescent NSs made from alternative materials possessing similar properties are highly desirable for overcoming the disadvantages and limitations of the QDs. In recent years, extensive research efforts have been devoted to the fluorescent NSs, which led to the fabrication of different types of nanoparticles such as fluorescent polymer nanoparticles, fluorescent silica composite nanoparticles, and lanthanide chelated hybrid nanoparticles [4,5]. In 2006, a new class of carbon NSs called carbon dots were first synthesised by Sun et al.; they exhibited multicoloured emissions of visible fluorescence in both solution and the solid state with a simple surface passivation method [6]. These carbon NSs possessed good fluorescent properties, such as low toxicity, tunable excitation and emission wavelength, good biocompatibility, high photostability, super antioxidant activity and bright electroluminescence [6,7,8,9,10]. Moreover, the main element of carbon NSs is carbon, which is not thought to be a toxic substance as compared to the heavy metals from conventional QDs. Therefore, the carbon NSs are expected to have great potential use in small active molecule detection, single optical fibres and nanosensors [11,12,13,14].

Up to now, many of kinds of methods, such as arc-discharge [15], electro-oxidation [16], laser ablation [6], hydrothermal route [17], nitric acid chemical oxidation [18,19] and microwave-hydrothermal synthesis [20,21,22], have been developed for the preparation of carbon NSs. The hydrothermal method involves carbonization, a complex pyrolytic reaction, including the processes of dehydrogenation, condensation, hydrogen transfer and isomerisation, which uses moderate temperatures and pressures over an aqueous solution of biomass for several hours [23]. Many raw materials including candle soot [24], plant soot [25], commercial activated carbon [18], lampblack [26], watermelon peel [27], small molecules [28], orange juice [29], etc., have been explored as raw materials for the synthesis of carbon NSs. We have reported the use of oligosaccharide cyclodextrin and polysaccharide cellulose to prepare multicolour carbon NSs [30]. They showed bright and colourful fluorescence in the whole visible spectral range, which was successfully used for mouse melanoma cell bio-imaging. These kinds of multicoloured NSs from renewable polysaccharide may have a great potential application in bio-molecular imaging.

In this work, the synthesis and characterization of multicoloured NSs were reported using starch and chitosan as raw materials. As linear polysaccharides, the starch and chitosan contain a large number of glucose or glucosamine units joined by glycosidic bonds. We compared the properties for these two kinds of polysaccharide-based NSs. The metal ion effects on the fluorescence intensity were investigated. The multicoloured NSs derived from starch were successfully used as multicoloured probes for the imaging of mouse melanoma cells. In addition, we further demonstrated that the polysaccharide NSs may also be used as unique nanostructural probes for the imaging of small aquatic craniate animals.

## 2. Results

### 2.1. Preparation of Polysaccharide-Based NSs

**Scheme 1:**
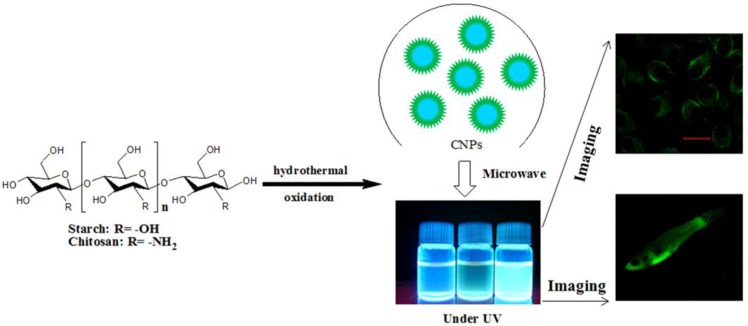
Synthesis fluorescent nanostructures (NSs) derived from starch and chitosan for bio-imaging.

The polysaccharide-based NSs were synthesised through a facile one-pot hydrothermal route using starch or chitosan as raw materials, respectively. As shown in Scheme 1, generally, polysaccharides of starch or chitosan underwent an alkali-assisted hydrothermal oxidation process to form the multicoloured NSs. We chose these two kinds of polysaccharides as raw materials for fluorescent NS preparation because they are both linear polymers with many glucose monosaccharide units. The literature about using different linear polysaccharides for multicoloured NS preparation to compare the differences between them is quite limited. The preparation of multicoloured NSs derived from starch or chitosan might provide new candidates for the development of novel polysaccharide-based fluorescent NSs. In a typical synthesis, starch (or chitosan) was dissolved in sodium hydroxide aqueous solution. The mixture in a stainless steel autoclave was hydrothermally heated at 160 °C for 4 h. After adjusting the pH value to 7, the obtained NSs were treated with a microwave for 5 min to improve the fluorescence properties. Redundant precursors and impurities were eliminated by filtration and the resulting NSs were freeze-dried for further use.

### 2.2. TEM Characterization of Polysaccharide-Based NSs

**Figure 1:**
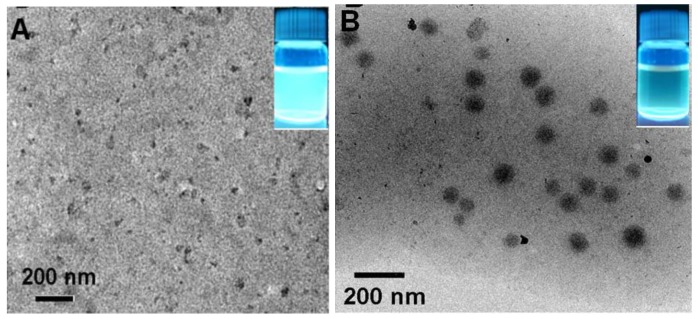
Transmission electron microscopy (TEM) images of polysaccharide-based NSs prepared from starch (**A**) and chitosan (**B**). Insets show the fluorescence photographs for the polysaccharide-based NSs in aqueous solution. Excitation wavelength for each sample in the inset pictures is 312 nm.

Figure 1 shows the TEM images of NSs prepared from starch and chitosan, respectively. It can be seen that that the polysaccharides, starch and chitosan, easily formed individual nanostructure under the treatment of alkali at 160 °C. This process involved the hydrothermal carbonization of the major constituents of sugars. As shown in Figure 1A, the starch NSs prepared by this method are irregular in shape with an average particle size of around 14 nm. However, the average diameter of chitosan NSs is about 75 nm, which showed a spherical morphology (Table 1). The molecular weight of starch is similar to that of the chitosan (~30 KDa). The starch is formed with a long chain of many of glucose units with hydroxyl groups, while the chitosan possesses amino groups. The different sizes of the polysaccharide-based NSs might be due to the different molecular structures of the starting materials. The resulting polysaccharide-based NSs are strongly fluorescent under the excitation of UV light (Figure 1). Both of them are well-dispersed in aqueous solution.

### 2.3. FT-IR Characterization of Polysaccharide-Based NSs

**Figure 2:**
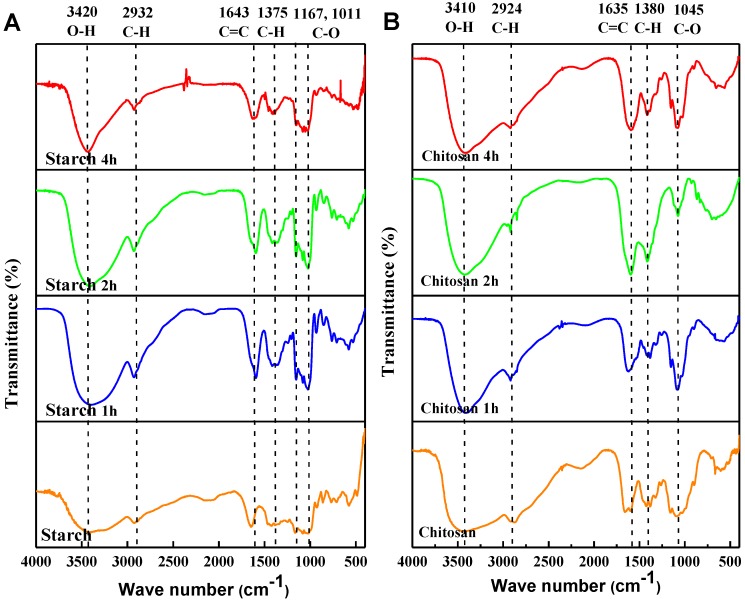
Fourier transform infrared (FT-IR) spectra of polysaccharide-based NSs prepared from (**A**) starch, starch NSs at 1, 2, 4 h, and (**B**) chitosan, chitosan NSs at 1, 2, 4 h.

Fourier transform infrared (FT-IR) spectra in Figure 2 reveal that the polysaccharide-based NSs have an arresting absorption peak in the range of 3410–3420 cm^−1^ corresponding to the stretching vibration of O–H or amino groups. Therefore, the presence of a large number of functionalised groups, such as hydroxyl or amino groups, not only leads to the good water solubility of NSs, but also makes the surface modification easier and more convenient. The peaks at 2924–2932 cm^−1^ correspond to the C–H vibrations of methylene. Moreover, there is an absorption peak at about 1635–1643 cm^−1^, which might be due to the vibrations of carboxyl groups and aromatic C=C structures. The absorption peaks at 1375 to 1380 cm^−1^ demonstrate the presence of the respective –CH_3_. Unlike the spectrum of chitosan, a strong absorption peak in the range of 1011–1167 cm^−1^ is observed, thus indicating the presence of a large number of hydroxyl groups for the starch NSs, while only weak absorption peaks at 1045 cm^−1^ corresponding to the C–O stretching vibration are found for the chitosan NSs. As for starch NSs, the peaks of the FT-IR remained unchanged with different degrees of carbonization by varying the reaction time from 0 to 4 h, while the peak at 1660 cm^−1^ for chitosan NSs fades away with the increased reaction time from 0 to 2 h, indicating the formation of amide groups. The zeta-potential measurement indicated that the starch NSs had a negative value less than −18 mV at pH 6.5, and a positive value of 17.5 mV for chitosan NSs (Table 1).

### 2.4. Fluorescence Properties Analysis of Polysaccharide-Based NSs

**Figure 3:**
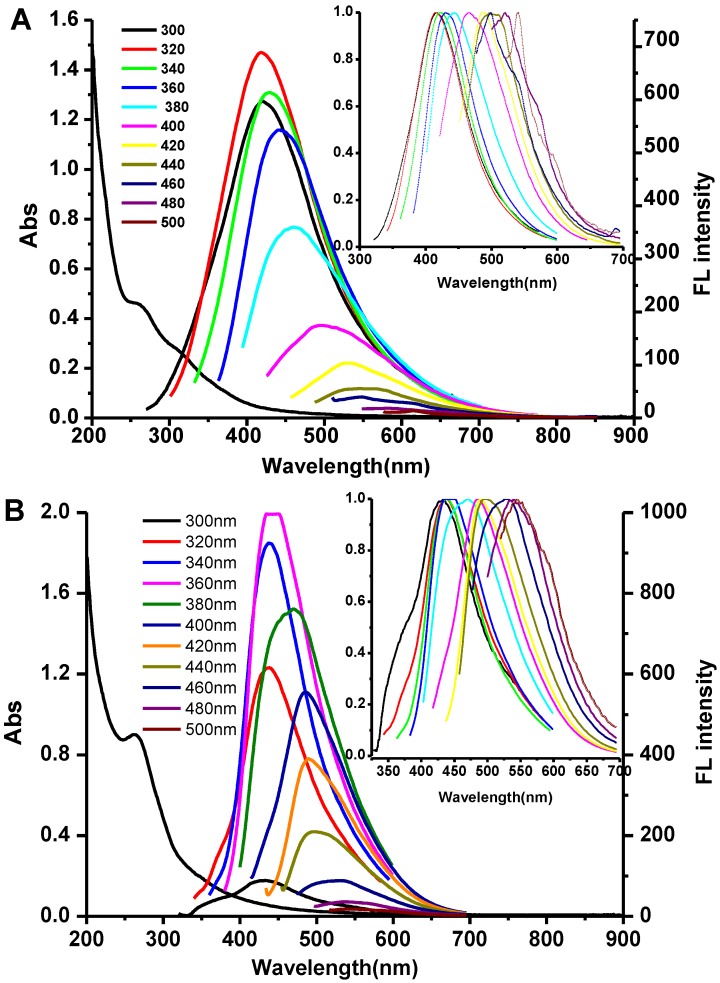
Ultra-violet visible absorption (Abs) and fluorescence (FL) emission spectra of polysaccharide-based NSs derived from starch (**A**) and chitosan (**B**). Excitation wavelengths were changed from 300 nm to 460 nm in 20 nm increments. Insets show the normalised emission spectra red-shifting with the excitation at longer wavelengths.

Figure 3 shows the fluorescence (FL) emissions and ultra-violet visible (UV/Vis) absorption spectra of polysaccharide-based NSs derived from starch (Figure 3A) and chitosan (Figure 3B). A broad peak at 275 nm in the UV/Vis absorption spectra was found for both of the polysaccharide-based NSs, which might come from the n-π* transition as reported in a previous work [31]. Under the effect of the excitation light with different wavelengths (300–500 nm), the light was emitted with different wavelengths mainly in the visible range, suggesting that the polysaccharide-based NSs exhibited an excitation-dependent emission phenomenon. With increasing excitation wavelengths, emission wavelengths also increase and the NSs emit at longer wavelengths (inset of Figure 3). The starch NSs under 320 nm excitation light show the strongest emission peak (Figure 3A) at 420 nm, and the chitosan NSs display similar properties at the 360 nm excitation light (Figure 3B), with a maximum emission at 445 nm (Table 1). The full width at a half maximum (FWHMs) for the polysaccharide-based NSs derived from starch and chitosan are 146 and 110 nm, respectively (Table 1). The size distributions of starch and chitosan NSs are in the range of 8–41 nm and 62–85 nm, respectively. It can be seen that the narrow size distribution corresponds to the smaller FWHMs, which was also observed in CdTe quantum dots [32]. The excitation-dependent fluorescence behaviour of the NSs might be ascribed to the effect of different surface states from different particle sizes and distributions. The TEM results demonstrated that the sizes of polysaccharide-based NSs are not uniform with a quite broad size distribution. Therefore, such excitation-dependent fluorescence characteristics might due to the different surface states resulting in the difference of the band gap of the NSs [33,34]. The quantum yield of starch NSs was 11.12% at the excitation wavelength of 360 nm, and 3.16% for the chitosan NSs.

### 2.5. Fluorescence Stability of Polysaccharide-Based NSs

**Figure 4:**
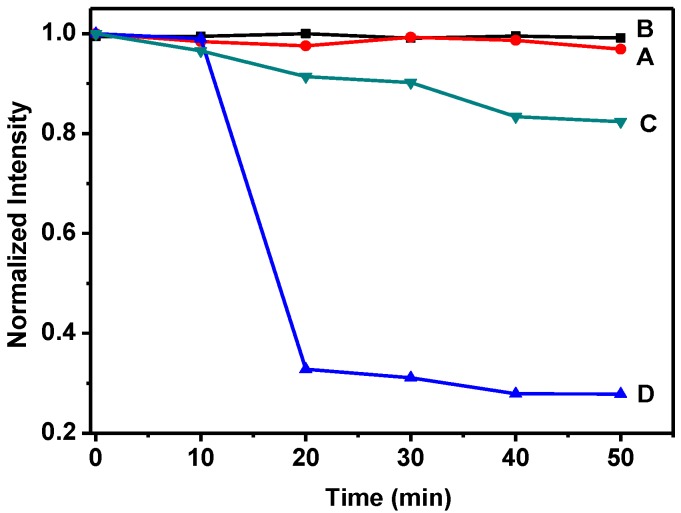
Photostability of the polysaccharide-based NSs prepared from starch (**A**) and chitosan (**B**), as compared with rhodamine B (**C**) and fluorescein (**D**).

Moreover, it is essential for the polysaccharide-based NSs to possess good photostability against bleaching when they are used as multicoloured agents in bio-imaging. As shown in Figure 4, the polysaccharide-based NSs derived from chitosan and starch showed excellent photostability in water as compared with the traditional fluorescent compounds rhodamine B or fluorescein. With the increase in test time, the fluorescence intensity of fluorescein plunged sharply within the first 20 min and decayed to 27% of the initial strength after 50 min. The fluorescence intensity of fluorescein decayed to 82% of the initial strength. However, the polysaccharide-based NSs exhibited excellent photostability with a decay of 4% and 1% for starch and chitosan, respectively. The super photostability of the polysaccharide-based NSs has great significance for their potential use in bio-imaging in harsh conditions.

### 2.6. Effect of Metal Ions on the FL Intensity of Polysaccharide-Based NSs

**Figure 5:**
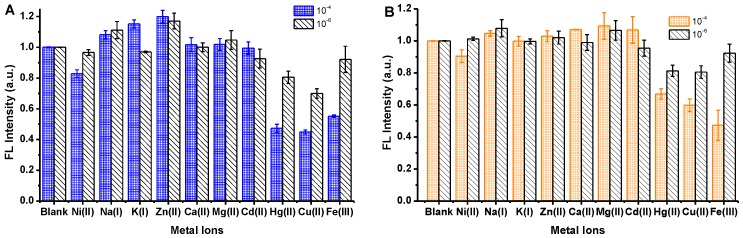
Effect of metal ions on the fluorescence (FL) intensity of the polysaccharide-based NSs prepared from starch (**A**) and chitosan (**B**).

The influence of metal ions on the FL intensity of the polysaccharide-based NSs is shown in Figure 5. It can be seen that 100 mΜ aqueous solutions of Cu(II), Hg(II) and Fe(III) exhibited a significant impact on the FL intensity of the NSs. About 40%–60% of the FL intensity value was reduced. However, an insignificant quenching effect was found for the equal amount of Ni(II), Na(I), K(I), Zn(II), Ca(II), Mg(II) and Cd(II) ions. Notably, Cu(II) resulted in the greatest reduction in the fluorescence intensity for the NSs derived from the starch (Figure 5A). However, Fe(III) showed the greatest quenching effect on the FL intensity of the NSs from chitosan, leading to a decrease in the fluorescence intensity of up to 40% (Figure 5B). The result is attributed to the fact that ions of Hg(II), Cu(II), and Fe(III) are oxidants; therefore, more electrons from the nanostructure surface are easily being caught by metal ions. Such an oxidation-reduction facilitated the recombination annihilation of non-radiative electrons/holes via an electron transfer process [35]. The effect of metal ions on the prepared NSs might have potential in fabricating turn-on smart sensors for highly sensitive metal ion detection.

### 2.7. pH Stability Experiments of Polysaccharide-Based NSs

**Figure 6:**
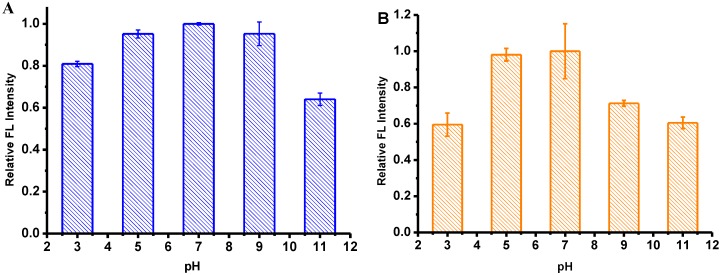
Effects of pH on the fluorescence (FL) intensity of the polysaccharide-based NSs derived from starch (**A**) and chitosan (**B**).

The pH effect on the FL intensity of the polysaccharide-based NSs was also investigated (Figure 6). Both of the multicoloured NSs showed the maximum fluorescence intensity at pH 7, while a significant decrease in the FL intensity (by 55%–81%) was observed upon changing from a neutral to either an acidic or a basic solution (at pH 3 or 11), which is consistent with that of the previous report [19]. The FL intensity is relatively stable at physiological pH range, revealing that the annihilation of the radiative recombination of energy-trapping on the NS surface was not significant. This might be due to the NSs being prepared in an alkaline environment via a complex carbonation reaction which may cause electrostatic doping/charging to the NSs and change their Fermi level [31,36]. Thus, the polysaccharide-based NSs might have potential bio-imaging applications in a wide physiological pH range [18].

### 2.8. Cytotoxicity Assay

**Figure 7:**
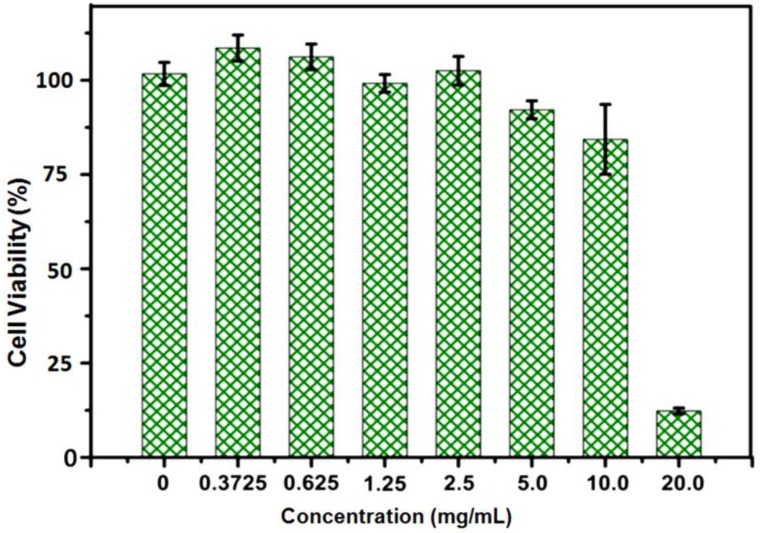
Cytotoxicity experiment of starch NSs. The values are the average of triplicate measurements.

Since the starch NSs possess relatively higher quantum yields, they would be more suitable for fluorescence cell imaging to gain a higher signal-to-noise ratio. In vitro cytocompatibility of the starch NSs with mouse melanoma cells was investigated by a MMT (3-(4,5-dimethyl-2-thiazolyl)-2,5-diphenyl-2-H-tetrazolium bromide)) assay. As shown in Figure 7, no significant reduction of cell viability was found when the concentration of NSs was less than 10 mg/mL for up to 24 h of exposure time. When the concentration of the starch NSs reached 20 mg/mL, the cell viability reduced dramatically to 12.5%. Therefore, they might not be considered harmful at a concentration less than 10 mg/mL for in vitro cell imaging applications.

### 2.9. In Vitro Tumour and Ex Vivo Guppy Fish Imaging

**Figure 8:**
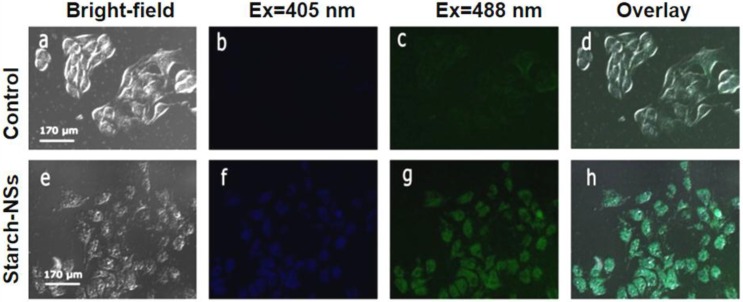
Laser scanning confocal microscopy images of mouse melanoma cells under bright-field, with excitation at 405 and 488 nm. The cells without polysaccharide-based NSs used as a control. Scale bar = 170 µm. (**a**) Bright-field image, fluorescence image by excitation at (**b**) 405 and (**c**) 488 nm, as well as (**d**) overlay of images of (**a**) and (**c**) for mouse melanoma cells without polysaccharide-based NSs. (**e**) Bright-field image, fluorescence image by excitation at (**f**) 405 and (**g**) 488 nm, as well as (**h**) overlay of images of (**e**) and (**g**) for mouse melanoma cells incubated with polysaccharide-based NSs.

**Figure 9:**
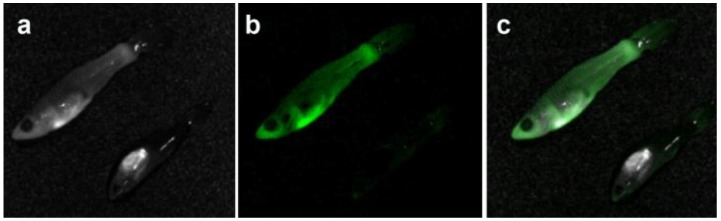
Ex vivo guppy fish imaging. Photograph of the starch NSs labelled guppy fish under (**a**) bright-field, (**b**) with excitation at 455 nm, and (**c**) overlay of (**a**) and (**b**) measured with CRi Meastro imaging system. Exposure time was 1500 ms. Small guppy fish in the bottom right corner was used as a control without being treated with the starch NSs.

The potential use of these starch NSs was explored with in vitro uptaking by mouse melanoma (B16-F10) cells. The B16-F10 cells were incubated with RPMI1640 medium containing the starch NSs for 12 h at 37 °C. Figure 8 shows the laser scanning confocal images of B16-F10 cells treated with the starch NSs at bright-field, excitation wavelengths of 405 nm and 488 nm, respectively. The B16-F10 cells treated with the polysaccharide-based NSs became bright at λ_ex_ = 405 and 488 nm, as compared to the control cells without the addition of NSs. This result demonstrated that the starch NSs entered into the cells resulting in multicoloured fluorescence, and multicoloured imaging due to the excitation-dependent emission property. Moreover, the starch NSs were also evaluated for organisms imaging by treating small guppy fish with these polysaccharide-based NSs. As shown in Figure 9, strong green fluorescence was observed from the big guppy fish as compared with small guppy fish (control). We selected guppy fish because they belong to small aquatic craniate animals, and the fluorescence signal can be detected with a Meastro in vivo imaging system. All these results demonstrated that the starch NSs might have potential for bio-imaging.

## 3. Materials and Methods

### 3.1. Materials and Instrumentation

Chitosan (molecular weight 30 KDa) was prepared through the degradation of raw material (Yuhuan Ocean Biomaterials Corporation, Zhejiang, China). Starch with a molecular weight 49k Da was purchased from Yufeng Starch products Co. Ltd. (Zhaoxian, China). Fluorescein and rhodamine B were purchased from Sinopharm Chemical Reagent Co. Ltd. (Shanghai, China). Quinine sulfate was purchased from Kermel Chemical Reagent Co. Ltd. (Tianjin, China). Mouse melanoma cell line B16-F10 was bought from KeyGen Biotechnology Co. Ltd. (Nanjing, China). Size-exclusion Sephadex gel G-25 was purchased from GE Healthcare (Fairfield, San Diego, CA, USA). Quinine sulphate, fluorescein and methyl thiazolyl tetrazolium (MTT) were ordered from Aladdin Reagent Co. Ltd (Shanghai, China). Dialysis bags with a molecular cut-off 3000 were purchased from Genestar Biotechnology Co. Ltd. (Shanghai, China). Muffle furnace was produced by Zhonghuan Experimental Electric Furnace Co. Ltd. (Tianjin, China). A JEOL model JEM-2000EX transmission electron microscope (TEM, Japan Electron Optics Laboratory Co. Ltd., Tokyo, Japan) was used to measure the shape and size of the NSs. Fourier transform infrared (FT-IR) spectra were measured using a VECTOR 22 FT-IR spectrometer (Bruker Co. Ltd., Fällanden, Switzerland) with a KBr pellets. A Perkin Elmer LS 55 spectrofluorometer was used for fluorescence spectra measurements. UV-Visible absorption spectra were measured on a Shimadzu UV2550 UV-Vis spectrophotometer (Shimadzu Co., Kyoto, Japan). FV 1000 confocal microscope (Olympus Corporation, Kyoto, Japan) was used for cell imaging experiment. Ex vivo imaging was carried out with a Cri Meastro Ex in vivo imaging system (Cambridge Research & Instrumentation, Inc, Woburn, MA, USA). Dimethylsulfoxide (DMSO) was bought from Damao Chemical Reagent Co. Ltd. (Tianjin, China). Sodium hydroxide (NaOH, >96%) was purchased from Dalu Chemical Reagent Co. Ltd. (Tianjin, China). Phosphate (H_3_PO_4_, >85%) was purchased from Hengxing Chemical Reagent Co. Ltd. (Tianjin, China). Boric acid (H_3_BO_3_, >99.5%) was purchased from Bodi Chemical Reagent Co. Ltd. (Tianjin, China). All other chemicals and reagents were purchased from local reagent store. The reagents were used without further purification unless otherwise stated.

### 3.2. Synthesis of Polysaccharide-Based NSs

The carbon sources of starch (1 g) or chitosan (1 g) and 0.1 g sodium hydroxide were added to 15 mL of deionised water with vigorous stirring. The reaction mixture was then put into a Teflon-lined stainless-steel autoclave and sealed heating at 160 °C for 4 h. Subsequently, the pH of the solution was adjusted to 7 using 1 M hydrochloric acid. The resulting NSs were thoroughly dialyzed for 48 h against distilled water through dialysis bags with the molecular weight cutoff of 3000. Further, the solution was centrifuged at a speed of 4000 rpm to remove impurities with larger molecules. Relatively small impurity was removed by microporous membrane made from mixed cellulose with a pore size of 0.22 μm. The obtained solution of polysaccharide-based NSs was then heated with a microwave oven (800 W) for 5 min. Later, the solution was cooled and purified with sephadex G-25 column. The fluorescent components were collected, freeze dried and stored at 4 °C for further characterization and use.

### 3.3. Characterization of Polysaccharide-Based NSs

NSs ethanol solution (2 mg/mL) was dropped onto 400-mesh Carbon-coated Cu grids for TEM analysis. FT-IR spectroscopy measurement was performed on a VECTOR 22 spectrometer (Bruker Co. Ltd., Fällanden, Switzerland) with KBr pellets (samples/KBr 1:100, *wt*/*wt*) to analyse the surface groups of NSs. Fluorescence quantum yield (Φ) of the NSs was calculated according to the following equation [34]:

Φ_1_ = Φ_2_*I*_1_*A*_2_η^2^_1_/*I*_2_*A*_1_η^2^_2_ (1)

where *I*_1_ and *I*_2_ are the fluorescence intensities of the NSs and the standard quinine sulfate, and *A*_1_ and *A*_2_ represent the optical densities of the NSs and the standard, respectively; η_1_ and η_2_ are the refractive index of the NSs and the standard, respectively. The Φ_2_ for quinine sulfate at 360 nm was 0.54 in 0.1 M H_2_SO_4_ (refractive index: 1.2). The NSs were dissolved in water (refractive index: 1.2). In order to minimise the reabsorption effects, absorbance values of the measured solutions were kept less than 0.1 at the excitation wavelength. Both the excitation and emission slit widths were 5 nm during the FL spectra measurement.

### 3.4. Photostability Study of Polysaccharide-Based NSs

The photostability experiment of the polysaccharide-based NSs (1 mg/mL), rhodamine B and fluorescein in distilled water against photo-bleaching was performed under a 40 W incandescent lamp as an excitation source. The multicolour NSs, rhodamine B or fluorescein were added to a 10 mm fluorescence cuvette and kept 10 cm away from the excitation lamp. The fluorescence intensity of the multicolour NSs was measured with spectrofluorometer every 10 min.

### 3.5. Metal Ions Effects on FL Intensity

MgCl_2_·6H_2_O, CaCl_2_, NaCl, CuSO_4_·5H_2_O, K_2_CO_3_, ZnCl_2_, NiSO_4_·6H_2_O, FeCl_3_, Hg_2_Cl_2_ and CdCl_2_ were added to distilled water to prepare standard solutions (10^−4^ mol/L or 10^−6^ mol/L). Then 0.5 mL of NSs aqueous solution (0.5 mg/mL) was added to 4.5 mL of standard metal ions solution and stirred at room temperature for 30 min. Then, the FL intensity of mixture solution was measured with LS55 fluorescence spectrofluorometer (Perkin-Elmer) at the emission wavelength of 450 nm. The FL intensity of the polysaccharide-based NSs was normalised and plotted as the mean values of three measurements ± SD (standard deviation).

### 3.6. pH Effect on FL Intensity of the Polysaccharide-Based NSs

Britton-Robinson buffer containing 0.04 mol/L H_3_BO_3_, 0.04 mol/L H_3_PO_4_, 0.04 mol/L CH_3_COOH was used for the study of pH effect with variable range of pH 3 to pH 11. Various pH (pH 3–11) solutions were made by adding certain amount of 0.2 mol/L of sodium hydroxide solution. Then 200 μL of polysaccharide-based NSs solution (1 mg/mL) was added to 2 mL of Britton-Robinson buffer, and the FL intensity at 450 nm was measured with fluorescence spectrometer with a 320 nm excitation wavelength and 1200 nm/min scan rate.

### 3.7. Cytotoxicity Assay of the Polysaccharide-Based NSs

In vitro cytocompatibility of the NSs with mouse melanoma (B16-F10) cells was investigated by seeding the cells (5 × 10^3^ cells/well) in a 96-well plates containing RPMI-1640 medium supplemented with 10% fetal bovine serum and 1% antibiotic-antimycotic solution. The plates were maintained at 37 °C in a humidified 5% CO_2_ atmosphere. After adding various concentrations (0–20 mg/mL, *n* = 3) of NSs, the cells were cultured for another 24 h, and 20 μL of MTT (5 mg/mL) solution was added to each well. The cells were further incubated for 4 h and washed with PBS for three times. After adding 100 μL of DMSO, the optical density (OD) of the solution was recorded by a Microplate Reader (Wellscan MK3, Labsystems, Helsinki, Finland) at 570 nm. The cell viability was calculated by using the equation: Cell Viability [%] = (OD_treated_/OD_control_) × 100% (OD_control_ is measured in the absence of agent, and OD_treated_ donates the intensity obtained in the presence of NSs).

### 3.8. In Vitro Mouse Melanoma Cell Imaging

B16-F10 mouse melanoma cells were seeded in 24-well tissue culture plates with a glass slide at the bottom at a density of 3 × 10^4^ cells/well at 37 °C in a humidified 5% CO_2_ atmosphere. 100 µL multicoloured starch NSs (3.8 mg/mL) and 400 µL medium were added to each well. The glass slide with cells was washed thoroughly with PBS buffer after 5 h incubation, and the cells were fixed with formalin. In vitro tumour cell imaging was conducted with a FV 1000 laser confocal microscope and the fluorescence images were collected in blue and green region with exposure time 100 ms.

### 3.9. Ex Vivo Guppy Fish Imaging

Guppy fish were put in the water mixed with the starch NSs (~5 mg/mL) and the control fish were put in fresh water. After 10 min, the fish were thoroughly washed with distilled water and taken image on a CRi Meastro Ex in vivo imaging system. Spectral fluorescence images could be produced by selecting appropriate filters for NSs with excitation 455 nm, emission 515 nm long-pass filter, acquisition settings 500–750 nm in 10 nm steps). In this study, the exposure times were automatically calculated and1500 ms was selected.

## 4. Conclusions

In summary, we demonstrated a facile one-pot hydrothermal method for the preparation of polysaccharide-based NSs using starch or chitosan as raw materials. The obtained NSs are nano-sized and highly water-soluble due to the presence of hydroxyl or amino groups on the surface for starch and chitosan NSs, respectively. Under ultraviolet excitation, the NSs exhibited strong fluorescence with an excitation-dependent emission behaviour. The polysaccharide-based NSs are very stable against photo-bleaching as compared with the traditional fluorescent compounds. Some oxidative metal ions, Hg(II), Cu(II), and Fe(III), displayed a quenching effect on the FL intensity of the NSs. The NSs showed a maximum FL intensity at physiological pH. The cytotoxicity study demonstrated that the starch NSs are safe for B16-F10 cells at a concentration less than 10 mg/mL. The multicoloured starch NSs showed success as fluorescent probes for B16-F10 melanoma cells and guppy fish imaging. The results of this work represent a step toward the design of polysaccharide-based NSs for bio-imaging applications.

## Figures and Tables

**Table 1:** Physicochemical parameters of the polysaccharide-based novel nanostructures (NSs) prepared from starch and chitosan.

Sample	FWHM ^1^ (nm)	Size Distribution (nm)	Max Em ^2^ (nm)	Zeta Potential (mV)	QY ^3^ (Φ*, %*)
Starch NSs	146	8–41	420	−18.0	11.12
Chitosan NSs	110	62–85	445	+17.5	3.16

^1^ FWHM, the full width at a half maximum; ^2^ Em, emission wavelength; ^3^ QY, Quantum yield at 360 nm.

## Abbreviations

The following abbreviations are used in this manuscript:

NSs	nanostructures
QDs	quantum dots
UV	ultra-violet
FL	fluorescence
FWHM	the full width at a half maximum
Em	emission wavelength
QY	Quantum yield at 360 nm
Abs	absorption
DMSO	Dimethylsulfoxide
TEM	transmission electron microscope
FT-IR	Fourier transform infrared
MMT	3-(4,5-dimethyl-2-thiazolyl)-2,5-diphenyl-2-H-tetrazolium bromide
